# Supplementary material for: Phytochemicals in Chinese Chive (Allium tuberosum) Induce the Skeletal Muscle Cell Proliferation via PI3K/Akt/mTOR and Smad Pathways in C2C12 Cells
Source: Int J Mol Sci. 2021 Feb 25;22(5):2296. doi: 10.3390/ijms22052296 (PMC7956299; doi:10.3390/ijms22052296)
Supplement: Supplementary file 1 [file ijms-22-02296-s001.pdf]

Supplementary Materials

Phytochemicals in Chinese chive (*Allium tuberosum*) Induce the Skeletal Muscle Cell Proliferation via PI3K/Akt/mTOR and Smad Pathways in C2C12 Cells

Mira Oh[a, †], Seo-Young Kim[b, †], SeonJu Park[b], Kil-Nam Kim[b,*], and Seung Hyun Kim[a,*]

[a] *College of pharmacy, Yonsei Institute of Pharmaceutical Sciences, Yonsei University, Incheon, Korea*

[b] *Chuncheon Center, Korea Basic Science Institute (KBSI), Chuncheon, 24341, Republic of Korea*

[†] *These authors contributed equally to this work*

[*]Corresponding author

Kil-Nam Kim

Tel : +82-33-815-4607

Fax : +82-33-255-7273

E-mail : knkim@kbsi.re.kr

[*]Corresponding author

Seung Hyun Kim

Tel : +82-32-749-4514

Fax : +82-32-749-4105

E-mail : kimsh11@yonsei.ac.kr

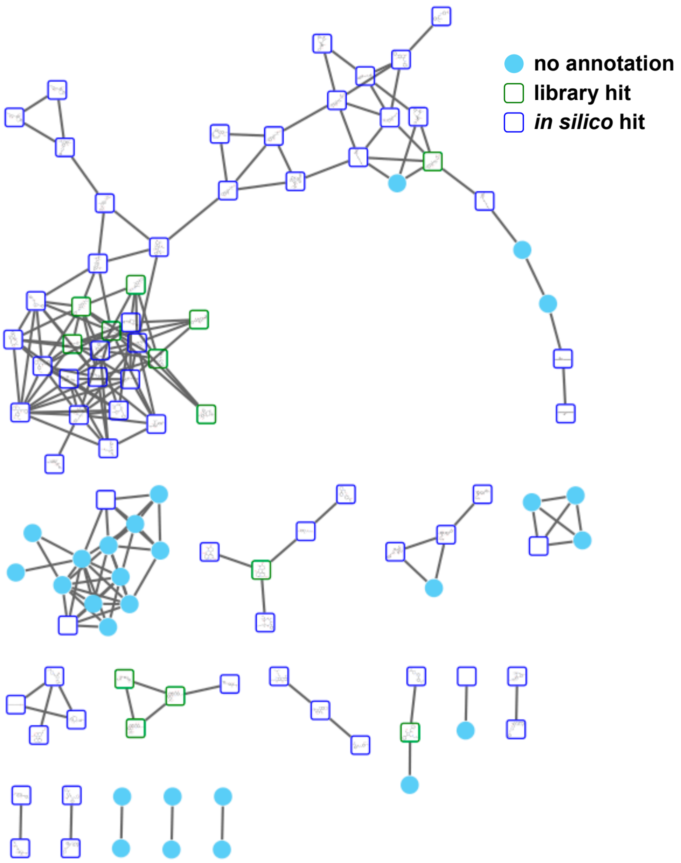

Figure S1. The networking analysis of Chinese chive by NAP. Network was visualized with nodes (compounds) and edges (mass differences) through Cytoscape 3.8.0. Blue hollow nodes are represented by NAP Consensus top ranked candidates annotated *in silico*.

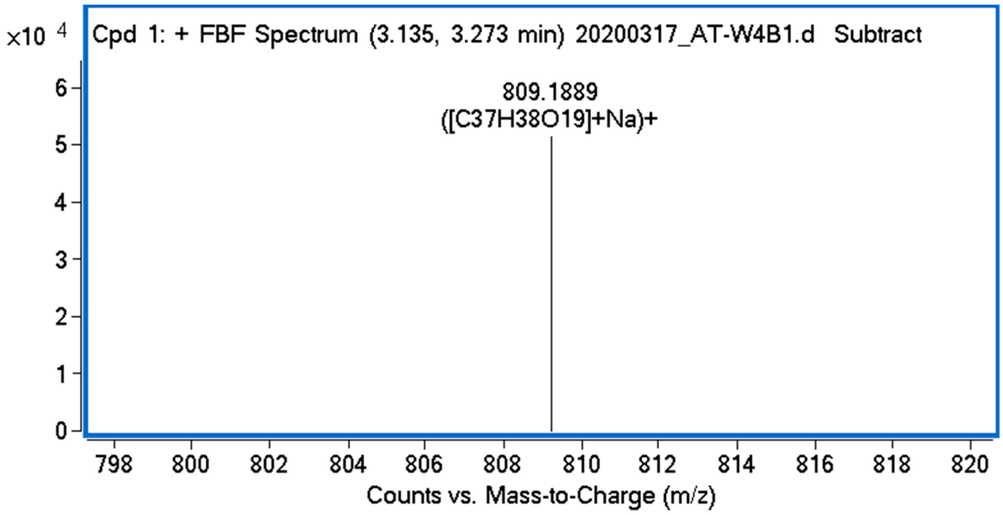

Figure S2. HR-ESI-MS of compound **1.**

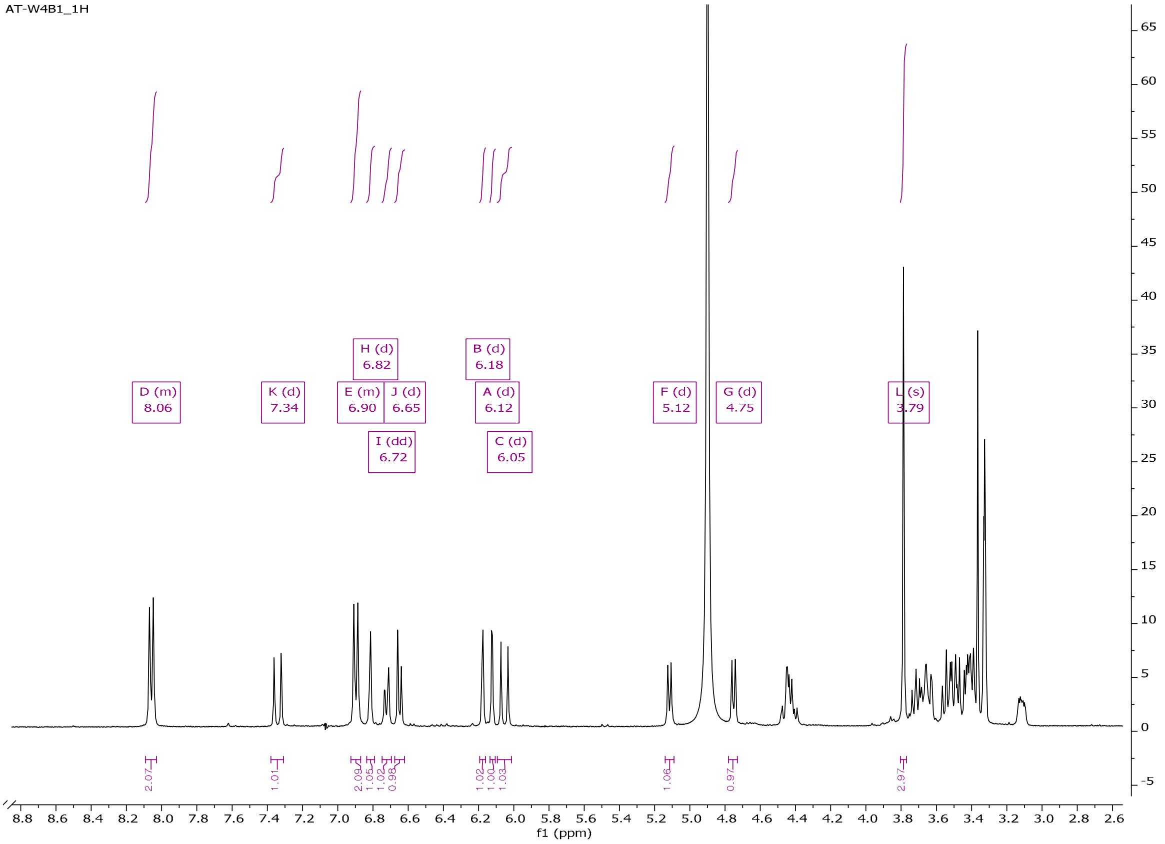

Figure S3. ^{1}H-NMR spectrum of compound **1** (400 MHz, methanol-d_4)

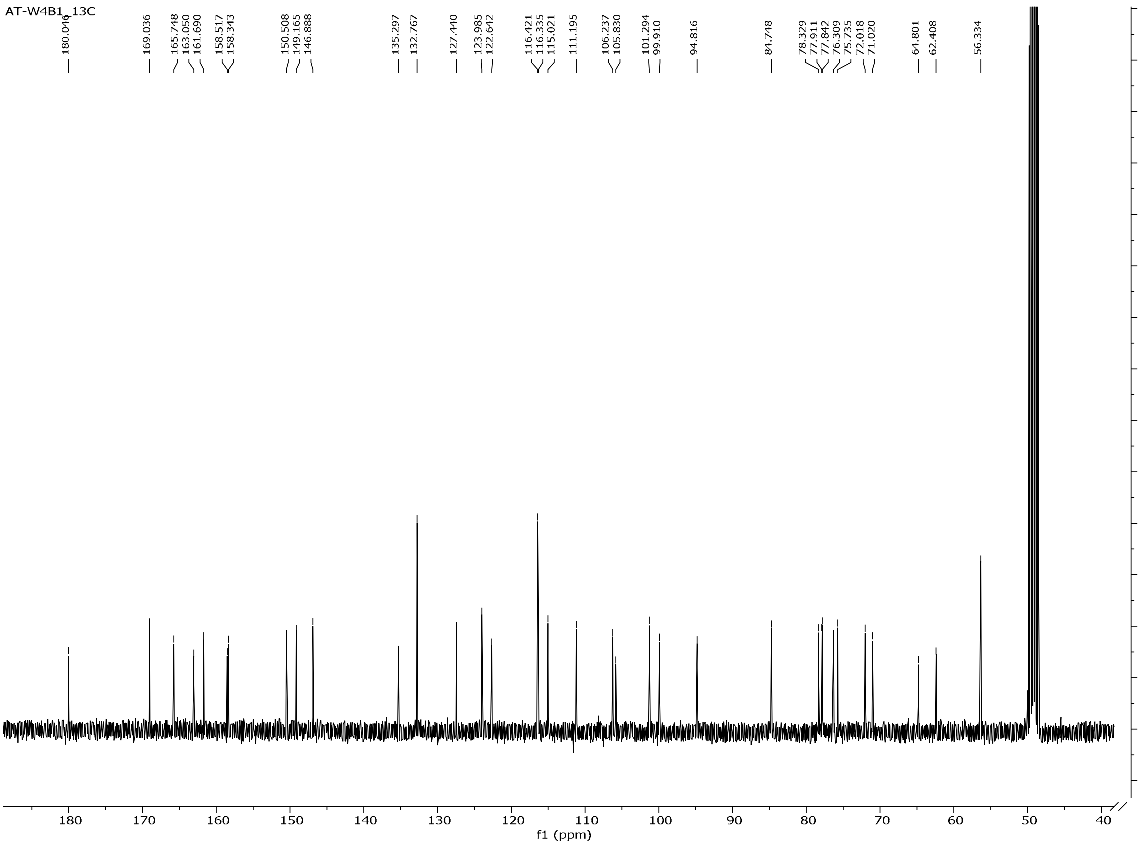

Figure S4. ^{13}C-NMR spectrum of compound **1** (100 MHz, methanol-d_4)

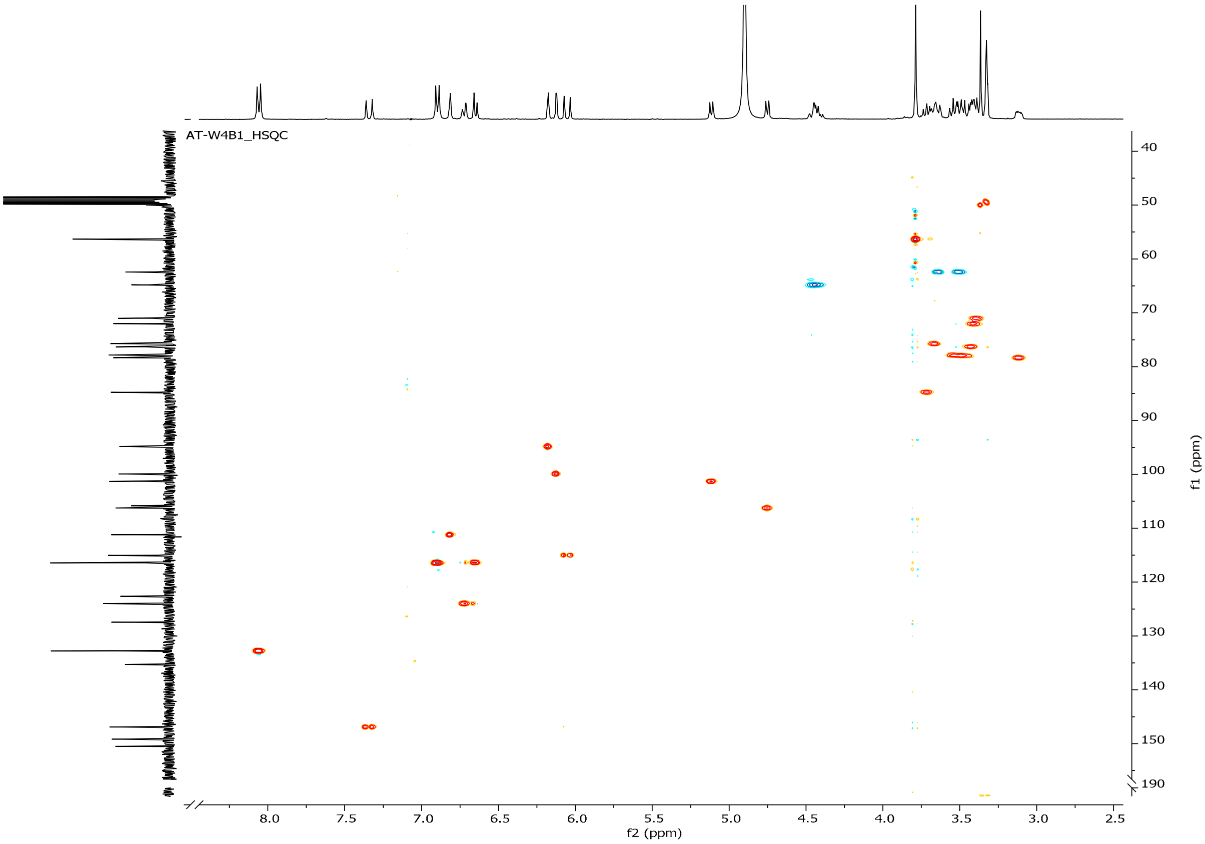

Figure S5. HSQC spectrum of compound **1** (methanol-d_4)

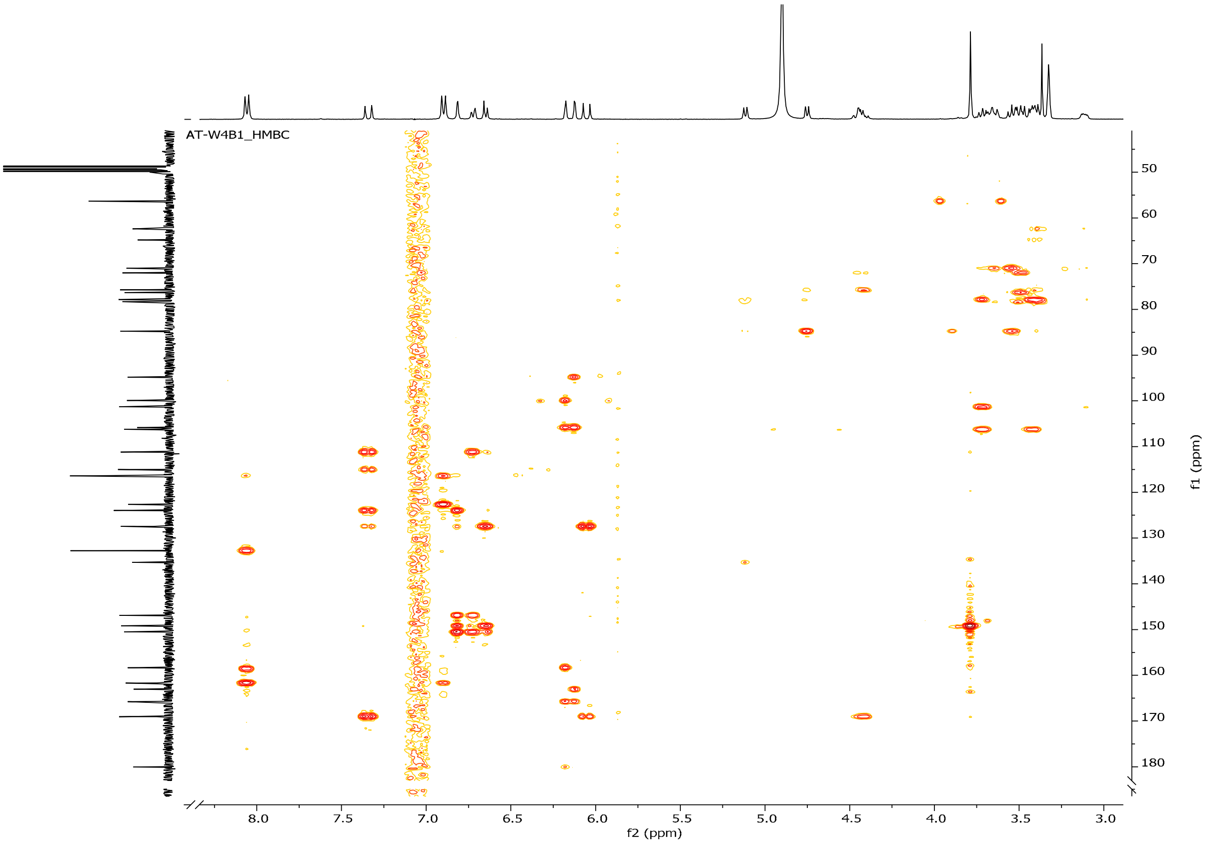

Figure S6. HMBC spectrum of compound **1** (methanol-d_4)

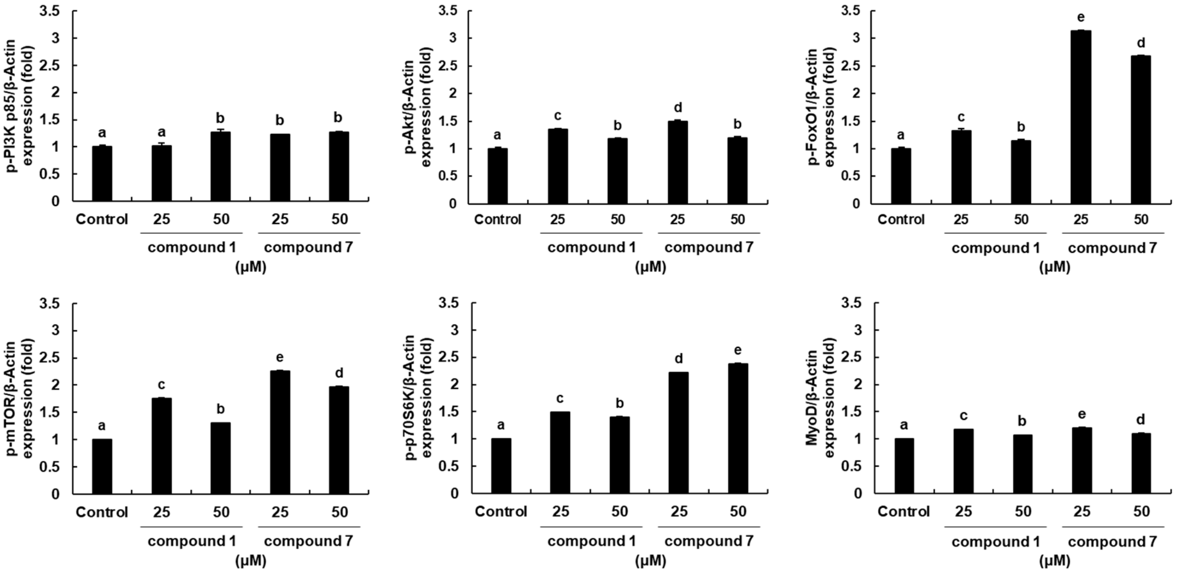

Figure S7. Skeletal muscle cell differentiation activities of compound **1** and **7** from Chinese chive on C2C12 cells. The levels of p-PI3K p85, p-Akt, p-FoxO1, p-mTOR, p-p70S6K, MyoD proteins were determined using Western blot analysis. Every level of proteins versus β-Actin were measured by densitometry. Data were statistically compared by two-tailed one-way ANOVA and Tukey's post-test using Prism software (Version 4.00; GraphPad Inc., La Jolla, CA, USA). $^{a-e}$Values having different superscripts are significantly different at $p < 0.05$.
